# Data integration for inference about spatial processes: A model-based approach to test and account for data inconsistency

**DOI:** 10.1371/journal.pone.0185588

**Published:** 2017-10-03

**Authors:** Simone Tenan, Paolo Pedrini, Natalia Bragalanti, Claudio Groff, Chris Sutherland

**Affiliations:** 1 Vertebrate Zoology Section, MUSE - Museo delle Scienze, Corso del Lavoro e della Scienza 3, 38122 Trento, Italy; 2 Provincia Autonoma di Trento, Servizio Foreste e Fauna, Via Trener 3, 38100 Trento, Italy; 3 Department of Environmental Conservation, University of Massachusetts, Amherst, MA, 01003, United States of America; Universita degli Studi di Trento, ITALY

## Abstract

Recently-developed methods that integrate multiple data sources arising from the same ecological processes have typically utilized structured data from well-defined sampling protocols (e.g., capture-recapture and telemetry). Despite this new methodological focus, the value of opportunistic data for improving inference about spatial ecological processes is unclear and, perhaps more importantly, no procedures are available to formally test whether parameter estimates are consistent across data sources and whether they are suitable for integration. Using data collected on the reintroduced brown bear population in the Italian Alps, a population of conservation importance, we combined data from three sources: traditional spatial capture-recapture data, telemetry data, and opportunistic data. We developed a fully integrated spatial capture-recapture (SCR) model that included a model-based test for data consistency to first compare model estimates using different combinations of data, and then, by acknowledging data-type differences, evaluate parameter consistency. We demonstrate that opportunistic data lend itself naturally to integration within the SCR framework and highlight the value of opportunistic data for improving inference about space use and population size. This is particularly relevant in studies of rare or elusive species, where the number of spatial encounters is usually small and where additional observations are of high value. In addition, our results highlight the importance of testing and accounting for inconsistencies in spatial information from structured and unstructured data so as to avoid the risk of spurious or averaged estimates of space use and consequently, of population size. Our work supports the use of a single modeling framework to combine spatially-referenced data while also accounting for parameter consistency.

## Introduction

Obtaining precise estimates of population density and space use can lead to a better understanding of the processes governing spatiotemporal ecological dynamics and, in turn, improve wildlife management and conservation practices. The task of estimating ecological state variables is, however, challenging, especially for rare or elusive species such as large carnivores, and requires analytical approaches that account for the fact that not all individuals in a population can be observed [1]. Regardless of methodology, the quality of model-based inference is directly related to data quality, which can be an issue for elusive species, especially when resources for monitoring are limited. This has led to an emphasis on developing methods that integrate multiple data sources [2, 3] and, importantly, to a realization that the vast amounts of data regularly collected outside of formal scientific studies, *unstructured* or *opportunistic* data, are a potentially valuable data source [4, 5]. Although the majority of data integration methods have focused on improving estimates of species distribution and temporal population trends, opportunistic data has great potential to improve inferences about spatial ecological processes.

Integrated population models (IPMs: [2, 6, 7]) provide a statistical framework for jointly modeling count data and demographic data, typically resulting in improved inferences about the mechanisms regulating population dynamics. As a result, there has been continued development of more general ‘integrated data models’ that seek to combine any independent data sources that arise from the same ecological process [3]. For example, occupancy and abundance are two directly related ecological state variables, and joint analysis of capture-recapture and occupancy data has been shown to improve estimates of abundance [8, 9], density [10], and even colonization-extinction dynamics and dispersal [11]. A common feature of the majority of studies that use multiple data sources, aside from improving parameter precision, is that each independent data set is collected according to a well-defined sampling protocol, i.e., an integration of structured data sets. The value of integrated models that use unstructured or opportunistic data, such as that collected by many citizen scientists, is yet unclear. For example, van Strien et al. [12] argue that opportunistic data represents an important data source that, if analyzed appropriately, can yield improved inferences about temporal trends in occurrence, while Kamp et al. [13] caution against its use, demonstrating that citizen science data were unable to detect significant species declines. Regardless, with the rapid increase in citizen science initiatives, finding innovative ways to utilize opportunistic data will broaden the scope of ecological enquiry that can be addressed within a single analytical framework [3].

Spatial capture-recapture (SCR) methods [14–16] are now well-established in applied ecology and produce estimates of population density using spatial encounter history data. Using spatial patterns of observations to account for heterogeneity in detection due to differences in trap exposure (i.e. distance between traps and individual home ranges), and treating space as an explicit model component, SCR produces unbiased estimates of density and space use across a range of conditions (e.g. [15, 17–19]). Moreover, SCR has been used to estimate density for elusive species from data collected using a variety of field methodologies including camera traps [20], hair snares [21], and scat surveys [22]. A core component of SCR is an explicit model for space use that relates encounter probability to the distance from an individual’s activity center via the estimation of a spatial scale parameter *σ* [15]. Estimating *σ* yields an explicit definition of the effective sampling area, and as a result, absolute density can be directly estimated. If follows that to estimate density well, *σ* must also be well estimated. As with other statistical methods, the precision of SCR-derived estimates of space use and density depend on sample sizes, specifically, but not solely, the number of unique spatial locations that individuals are observed at (spatial recaptures). Thus, adding additional spatial information should, in theory, lead to improved inference about space use, and in turn, density. For example, Gopalaswamy et al. [23] increased the number of spatial recaptures by integrating camera trap and scat collection data which resulted in more precise estimates of density, while Royle et al. [24] and Sollmann et al. [25] demonstrated that space use (*σ*) and density are estimated with higher precision when telemetry data are used in addition to traditional capture-recapture data (See also Table 1).

**Table 1:** Summary of contributions that provide an integrated framework for spatially-referenced individual data. Systematic data are collected under specific study designs: spatial capture-recapture (SCR), telemetry, and counts or binary detections (survey). Parameter shared: *ψ*, Data Augmentation parameter; *σ*, scale parameter of the observation model; *ϕ*, survival probability; *α*, effect of a landscape covariate on the relative probability of use; *δ*, individual-level recruitment probability.

		Systematic			Opportunistic		
Paper		SCR	Telemetry	Survey		Parameter	Study species
Sollmann et al., 2013a	[25]	•^1^	-	-	-	*σ*	jaguar
Gopalaswamy et al., 2012	[23]	•^1^	-	-	-	*ψ*, *σ*	tiger
Sollmann et al., 2013b	[26]	•^2^	•	-	-	*σ*	raccoon
Sollmann et al., 2013c	[27]	•^2^	•	-	-	*σ*	Florida panther
Royle et al., 2013	[24]	•	•^3^	-	-	*σ*, *α*	black bear
Linden et al., 2017	[28]	•	•^3^	-	-	*σ*, *α*	American marten
Chandler et al., 2014	[10]	•	-	•	-	*ϕ*, *δ*	black bear
Present study		•	•	-	•	*σ*	brown bear

^1^ camera trapping and scat collection;

^2^ extended to mark-resight;

^3^ resource selection function data

Interestingly, in the context of SCR, telemetry data require no information about sampling effort because observed locations provide representative information only about the spatial scale parameter (*σ*), and thus any amount of telemetry data are likely to be informative about space use. So, while the inability to quantify observer effort and bias is often cited as a major limitation of data collected by citizen scientists [29], it appears that such opportunistic data lends itself naturally to integration within the SCR modeling framework. Specifically, when opportunistic observations can be made of individually-identifiable animals, i.e., via direct or indirect recognition of naturally marked, collared or tagged individuals or the collection of DNA yielding biological samples such as hair or faeces, the locations of those observations are informative about space use. As a consequence, opportunistic observations have the potential to improve estimates of spatial parameters in SCR, and have the added benefit of potentially increasing the geographic extent of monitoring studies significantly.

Although a joint analysis of SCR, telemetry and opportunistic data is possible, such an approach assumes that data sources with shared parameters are indeed informative of the same process. For example, estimating a single spatial scale parameter, *σ*, from multiple data sources requires that the ecological process, space use, gives rise to the same spatial distribution of observations, i.e., the parameter is consistent. Despite the increasing use of integrated modeling approaches, this issue has received relatively little attention. Popescu et al. [30] demonstrated that information on space use obtained from camera trap and telemetry data are in general agreement and advocate for integrating additional sources of information when investigating space use. There are cases where the process that gives rise to spatial observation patterns differ, particularly when using both direct and indirect observations, or when the data types are associated with specific behaviors, for example territory marking at tree rubs compared to larger scale foraging patterns. So, because we *can* integrate multiple data sources does not necessarily mean that we *should*, and there is a need to find ways of testing for consistency in wildlife data.

Here we analyze data from a reintroduced brown bear *Ursus arctos* population in the central Alps, one of the most populated regions to be occupied by brown bears [31, 32]. This is a region where bear-human interactions are highly probable and any perceived threat is considered a key factor in determining the success or failure of the reintroduction [33]. First, we describe the brown bear sampling design and data, the classical SCR model, and how opportunistic data can be formally incorporated into spatial capture-recapture methods. We then demonstrate an application of a model-based test for parameter consistency across data types using Bayesian variable selection.

## Materials and methods

This study used data from GPS collared brown bears which were captured and collared with the permission of the Italian Ministry of the Environment.

### Study area and population

This study was conducted in 2013 in the Italian Alps, an area characterized by a mosaic of natural and human-modified habitats, with a landscape fragmented by urban areas and roads. Elevation ranges from 65 m to more than 3900 m a.s.l., with submontane, montane and subalpine vegetation covering areas below 2000 m, and human population density concentrated below 1000 m [33]. Between 1999 and 2002, nine bears (three males and six females, 3–6 years old) were released in the area as part of a reintroduction project to establish a self-sustained population [33, 34]. At the time, the original brown bear population consisted of at least three animals, which were assumed to have died without any genetic exchange with the translocated bears and their progeny [35].

### Brown bear data

#### Non-invasive genetic sampling

**Fig 1:**
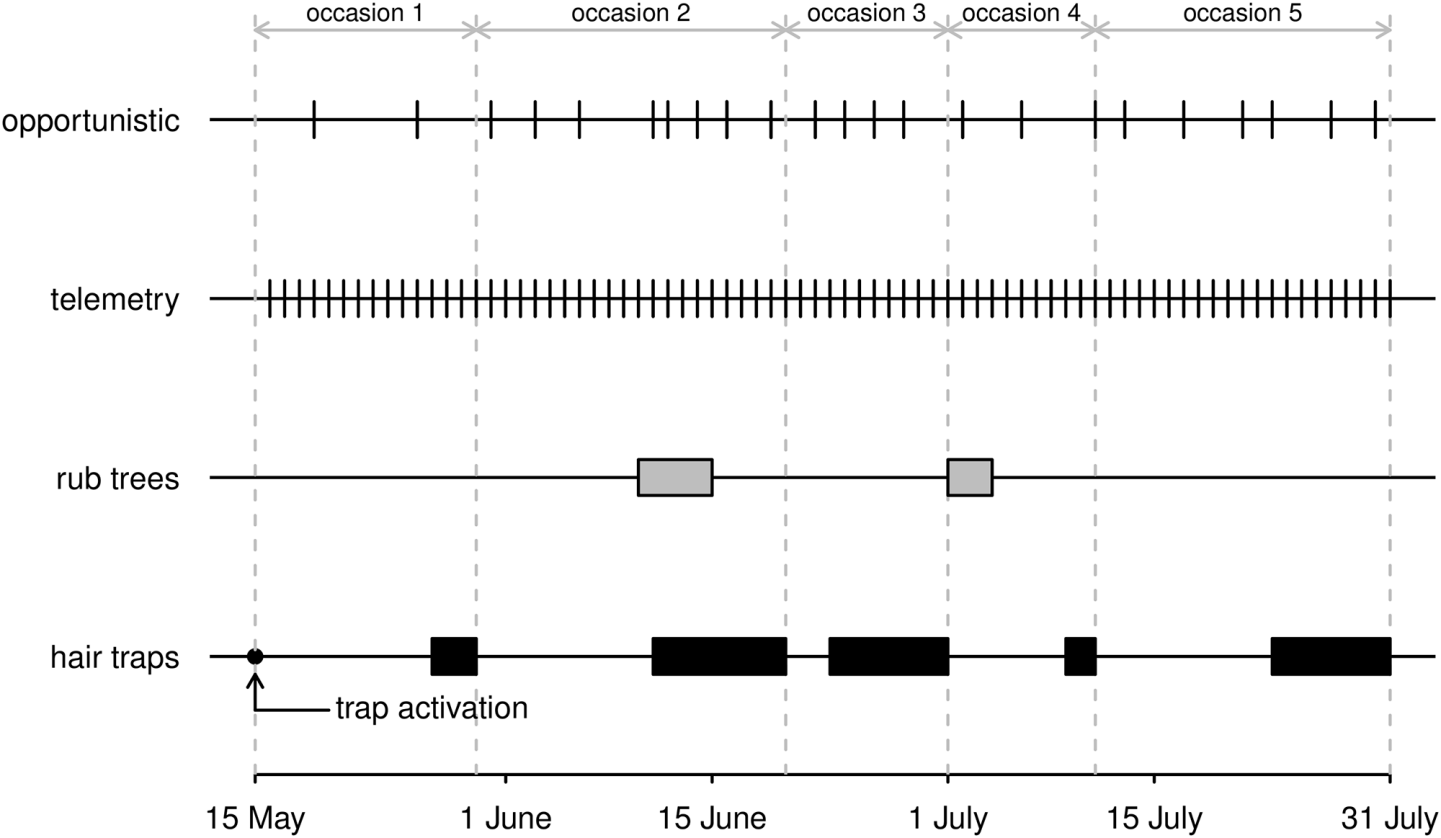
Timeline of data collection. The diagram shows the period when SCR data were systematically collected (i) using an array of hair traps checked on five occasions of variable length (black blocks), and (ii) from rub trees checked for hairs in two period (grey blocks). Telemetry data were thinned by randomly selecting one record per day, and opportunistic recovery of biological samples was performed in 23 days.

Bear hair samples were collected from 99 hair traps and 89 rub trees. Hair traps consisted of a strand of barbed wire wound around trees at *c*. 50 *cm* above ground level enclosing an area of *c*. 25 *m*^2^ with scent lure placed in the center [36]. They were set from 15 May to 31 July, checked on five occasions, and the number of days between occasions ranged from 3 to 10 days (Fig 1). Rub trees, barbed wire wrapped around trees, were monitored during the same period and were checked twice, first after six days and then after a further four days (Fig 1). All hairs on the hair trap and rub tree barbed wire were collected during each visit ensuring that only newly deposited hairs were collected in subsequent visits. Because the hair trap and rub tree data were collected according to a specific protocol, we refer to this structured data as traditional SCR data, or simply ‘SCR data’. In addition to the structured data collection, opportunistic hair and feces data were also collected [32, 35, 37, 38]. Following notification by third parties (typically members of the public), opportunistic sampling of hair and feces was carried out by agency personnel at sites where bear damage occurred, e.g. depredation on livestock, beehives and/or crops [38]. We refer to this data as ‘opportunistic data’.

**Fig 2:**
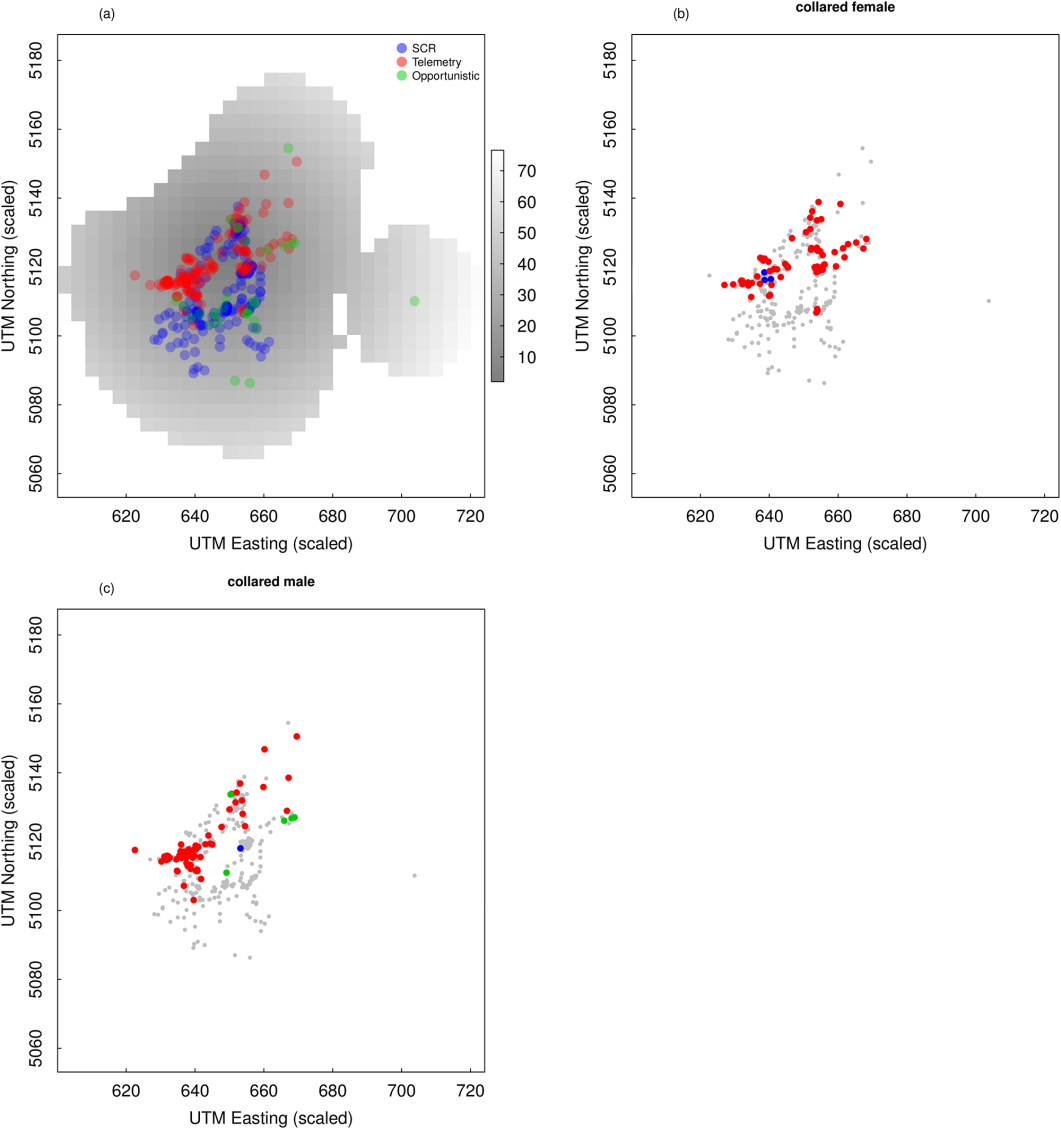
Spatial distribution of the three types of data available for the brown bear population in the central Alps. (a) Distance from the point were founders were released (in km) and location of bear captures from systematic sampling with hair traps and rub trees (SCR), telemetry and opportunistic records. (b-c) Location of the records for the two collared individuals from which telemetry information was derived. Grey dots indicate the location of all observed individuals.

Biological samples were genetically analyzed for individual identification. For a detailed description of DNA extraction methods, PCR protocols, protocols for individual identification, and molecular sexing, see [32, 35]. We considered only data belonging to the non-cub part of the population and successfully identified a total of *n* = 22 individuals (12 females and 10 males). Of the 22 individuals, 19 were detected using hair traps; two males and one female were sampled only on rub trees. During the period of trap deployment, 11 of the 22 individuals (four females and seven males) were detected opportunistically resulting in an additional 30 unique spatial locations (Figs 1 and 2a, S1 Fig).

#### Telemetry

Two bears, one male and one female, were tracked during the hair trap and rub tree sampling period using Global Positioning System (GPS) collars (Vectronic GPS-GSM collars, Vectronic Aerospace GmbH, Berlin, Germany). GPS collars collected positions at different intervals ranging from 10 min to 1 h. For the analysis we selected one random record per day per individual, giving a total of 143 unique telemetry locations (74 and 69 for the male and female respectively). The collared female was detected at a hair trap but never detected at rub trees or opportunistically (Fig 2b). The collared male was never detected with hair traps, but was detected once at a rub tree and was observed opportunistically five times (Fig 2c).

### Data analysis

#### Spatial capture-recapture data

Spatial capture recapture models are hierarchical models [39] that describe distance-dependent encounter probabilities (the observation process), and the spatial distribution of individuals across the landscape (density, the ecological state process). We adopt a Bayesian analysis of the model [21, 40] and assume that individual encounter data, *y*_*ijk*_, representing whether or not individual *i* was detected in trap *j* in occasion *k*, are Bernoulli random variables with success probability *p*_*ijk*_, i.e., the encounter probability:

$$\begin{array}{c} y_{ijk}∼\text{Bernoulli}\left(p_{ijk}\right). \end{array}$$ (1)

Encounter probabilities in SCR are assumed to decline with distance between a trap (*x*) and an individuals activity center (*s*) according to some decreasing function; here we use the half-normal encounter model and allow for sex-specific variation in the parameters:

$$\begin{array}{c} p_{ijk}=p_{0,ijk}\;\text{exp}(-\frac{1}{2\sigma_{\text{sex}}^{2}}\text{d}{\left(x_{j},s_{i}\right)}^{2}), \end{array}$$ (2)

where *σ*_sex_ is the sex-specific spatial scale parameter that determines the decrease in encounter probability with distance between trap *j* and individual *i*’s activity center (d(*x*_*j*_, *s*_*i*_)). The parameter *p*_0,*ijk*_ is the baseline encounter probability and can itself be modeled as a function of individual- (*i*), trap- (*j*) and occasion- (*k*) specific covariates. Specifically, we modeled the baseline encounter probability as a function of *sex*, trap type (*trap*: hair trap or rub tree), and, to account for the different time elapsed between consecutive sample occasions in each trap, time since last check (*time*), using standard logistic regression:

$$\begin{array}{c} \text{logit}\left(p_{0,ijk}\right)=\gamma_{0}+\gamma_{1}\;{\text{sex}}_{i}+\gamma_{2}\;{\text{trap}}_{j}+\gamma_{3}\;{\text{time}}_{jk}. \end{array}$$ (3)

where *γ*_0_ is the baseline encounter probability for females at hair traps, *γ*_1_ is the difference between male and female detections at hair traps, *γ*_2_ is the additive effect of trap type (i.e., the difference in detectability between hair traps and rub trees), and *γ*_3_ measures the change in detectability as time elapses since a trap was checked.

The second component of the SCR model is a point process model that describes the distribution of individual activity centers, *s*_*i*_, within a defined region, S, which should be large enough to contain all plausible activity centers of all observed individuals [15]. We were particularly interested in modeling density as a function of spatially varying covariates, i.e., as an inhomogeneous point process, and so we used a discrete representation of S defined as the center points of each pixel. In other words, the state-space S was defined as a raster, and individual activity centers (*s*_*i*_) were associated with pixel centroids. In our case, where we are considering a population that was established from a single release point, we modeled variation in density as a function of the distance from the point that the founding population was released between 1999 and 2002 (*d*.*release*; see ‘Study area and population’ and [41]). Using a binomial point process model, the per-pixel intensity, *μ*(*s*), is modeled as a log-linear function of ‘d.release’:

$$\begin{array}{c} \log\left(\mu \left(s_{g}\right)\right)=\beta_{0}+\beta_{1}\;{\text{d.release}}_{g}, \end{array}$$ (4)

for pixel *g* = 1, …, *nG*, with total number of pixels *nG* = 506, and the probability that an individual activity center is located in a pixel, *π*(*s*) is given by:

$$\begin{array}{c} \pi \left(s_{g}\right)=\frac{\mu (s_{g})}{\sum \mu (s_{g})}. \end{array}$$ (5)

This is the standard formulation of a Bayesian SCR model with a sex-specific half-normal encounter probability model, an inhomogeneous point process density model, and the estimation of sex-specific total population size *N* using data augmentation (see Chapters 7 and 10 in [15]). Sex was known for all observed individuals but not for unobserved (augmented) individuals so was modeled as an individual random effect to be estimated: sex_*i*_ ∼ Bern(*ω*_*sex*_), where *ω*_*sex*_ is the population-level sex ratio. We expected detectability to vary between sexes and trap type, and to be positively related to the time since last check. We also expected space use, *σ*, to vary by sex. Finally, we expected density to decline with distance from the release point.

The spatial encounter histories for the standard SCR analysis were generated for detections from a *J* = 188-trap array consisting of hair traps and rub trees across *K* = 5 sampling occasions (Fig 1 and S2 Fig). Data were formatted in a 3-dimensional *M* × *J* × *K* array, **Y**_*SCR*_, where *n* is the number of observed individuals and *M* − *n* is the number of augmented ‘all-zero’ encounter histories, a proportion of which are the estimated unobserved individuals. The additional data required to fit the model are: the coordinates of each hair trap and tree rub, a vector of sex determination of each individual, a *J* × *K* trap operation matrix which is a binary indicator denoting whether each trap was operational during sampling occasion, and the *J* × *K* matrix of ‘time since last check’ covariates, which were scaled to have zero mean and unit variance (S2 Fig).

#### Telemetry and opportunistic data

Unlike the traditional SCR data described above, both telemetry locations, **I**_tel_ and opportunistic data **I**_opp_ are not restricted to trap locations and therefore provide important additional information about individual movement, i.e., both are direct observations of space use [24]. We combine the individual telemetry and opportunistic locations and refer to them collectively as **I**_*i*_ for individual *i* = 1, …, *n*. These additional locations can be modeled using a bivariate normal ‘movement model’ with mean *s*_*i*_ and variance-covariance matrix Σ with variance $\sigma_{\text{sex}}^{2}$ and zero covariance [26, 27]:

$$\begin{array}{c} I_{i}∼BVN\left(s_{i},\Sigma \right) \end{array}$$ (6)

where,

$$\begin{array}{c} \Sigma =[\begin{array}{cc} \sigma_{\text{sex}}^{2} & 0\\ 0 & \sigma_{\text{sex}}^{2} \end{array}]. \end{array}$$ (7)

The parameters of this model can be related directly to the SCR half-normal encounter probability model (Eq 2) through the shared parameters *s* and *σ*, which means that telemetry data, opportunistic data and traditional SCR data can be jointly modeled, each contributing to the estimation of the latent activity centers and the spatial scale parameter *σ*.

The telemetry and opportunistic data, for *R*_tel_ = 143 and *R*_opp_ = 30 locations at which data were available, were formatted in two *R* × *n* × *K* arrays each, one containing x-coordinates and the other containing corresponding y-coordinates, for the *n* observed individuals and *K* occasions. This array structure allows the unstructured data (telemetry and opportunistic) to be related to the SCR data in the integrated model (S2 Fig). In addition, an *M* × *K* matrix denotes the number of unstructured locations for each individual in each occasion (S2 Fig).

In our case study telemetry data were available for two individuals only. We note, however, that the lack of this type of data is quite common in ecological and wildlife management studies (e.g. 3 collared individuals in [24] and [27]).

#### Model-based test for data consistency

First we evaluated the relative value of adding additional data sources by comparing estimates of density and space use obtained from the traditional SCR model with estimates obtained with the addition of telemetry data only, opportunistic sightings data only, and then both telemetry and opportunistic sightings data. Note that these models all assume consistency in the estimation of the spatial scale parameter *σ*_sex_ between structured (SCR) and unstructured (telemetry and opportunistic) data. To test the consistency assumption, we fit a fifth model to formally evaluate whether estimates of *σ* vary by data type. In summary, we fit the following five models:

1. Traditional SCR data only (data from hair traps and tree rubs)
2. SCR data + telemetry data (assuming data consistency)
3. SCR data + opportunistic sightings (assuming data consistency)
4. SCR data + telemetry + opportunistic sightings (assuming data consistency)
5. SCR data + telemetry + opportunistic sightings (data type-specific *σ*_type_).

Models 1 to 4 are described above. Model 5 uses a Gibbs variable selection approach (GVS: [42, 43]) to estimate the degree of support for the inclusion of a data type effect on *σ*, i.e., support for the hypothesis that estimates differ by data type. To do so, we formulate the model for *σ* as follows:

$$\begin{array}{c} \log\left(\sigma_{\text{type},\text{sex}}\right)=\theta_{\sigma }+\theta_{1}\;{\text{sex}}_{i}+w\;\theta_{2}\;\text{type} \end{array}$$ (8)

where *θ*_*σ*_ is the intercept of the log-linear model for *σ*, and because sex_*i*_ = 0 for female and type = 0 for traditional SCR data, exp(*θ*_*σ*_) is the female *σ* for structured data (*σ*_st,f_). The parameters *θ*_1_ and *θ*_2_ are the sex and data-type effects (differences) on *σ*, where estimates not overlapping 0 denote important effects. An important note is that the models that assume data consistency (models 2, 3, and 4) are special cases of this model but with *θ*_2_ fixed at 0. In model 5, the model where the difference between data types is examined, *θ*_2_ is an estimated parameter and is multiplied by an ‘inclusion parameter’ *w*, a latent binary variable with a Bernoulli prior distribution with probability 0.5. The posterior distribution of the inclusion parameter is the probability that there is a difference between types and therefore provides explicit inference about data-specific parameter consistency, and *θ*_2_|*w* = 1 is an estimate of the data type effect on *σ*.

For each model, we estimated sex-specific total population size, *N*_sex_, and sex-specific (and for model 5, data type-specific) spatial scale parameters, *σ*, and compared point estimates (posterior median) and precision (95% Bayesian Credible Interval width, BCI from here) of the estimated parameters. We adopted a Bayesian analysis of the SCR models using Markov chain Monte Carlo (MCMC) using the program JAGS [44] implemented in R [45]. In all models, we used an uninformative Normal(0, 100) priors for the parameters *γ* and *β* in Eqs 3 and 4. We used a Normal(0, 15) prior for the intercept of the log-linear model for sex- and data-specific *σ*, and a Uniform(-3, 3) prior for the sex and data type regression coefficients *θ*_1_ and *θ*_2_, respectively. In the model with variable selection to evaluate parameter consistency, a ‘slab and spike’ prior was used to improve the mixing and convergence time of the MCMC algorithm [42] (see S1 Text for details).

After testing a range of resolution values for the state-space, S, we used a resolution of 4 × 4 *km*, a value that was small enough to yield stable parameter estimates, and large enough to ensure the model was computationally tractable. To ensure the state space was large enough to contain all plausible activity centers, we used a 21 *km* buffer around the most extreme coordinates of all the data (telemetry, opportunistic and trapping data, Fig 2a). Data were augmented with *M* − *n* ‘all-zero’ encounter histories, where *M* = 300. Summaries of the posterior distribution were calculated from 30,000 post-burn-in posterior samples (burn-in = 3,000 iterations). The $\hat{R}$ diagnostics [46] used to assess convergence were < 1.02 for all parameters. The code for the fully-integrated models where (i) data consistency is assumed, (ii) data consistency is tested, or (iii) data inconsistency is accounted for, is available in S2 Text.

## Results

Estimates of the parameter relating density to distance to the reintroduction point (*β*_1_) were negative under all models, and although there was some variation in the strength of the effect, this result supports the hypothesis that density decreased with distance from the point were founders were released (S1 Table). The estimated sex ratio in the population, *ω*_*sex*_, did not vary significantly between the five models based on 95% Bayesian Credible Intervals and was lowest in SCR-only model (0.22, BCI: 0.09–0.41) and highest in the SCR + telemetry model (0.36, BCI: 0.19–0.57). Across all models, detectability was higher for males, higher at hair traps, and increased with increasing time between checks (S1 Table).

### Assuming data consistency in the model

**Fig 3:**
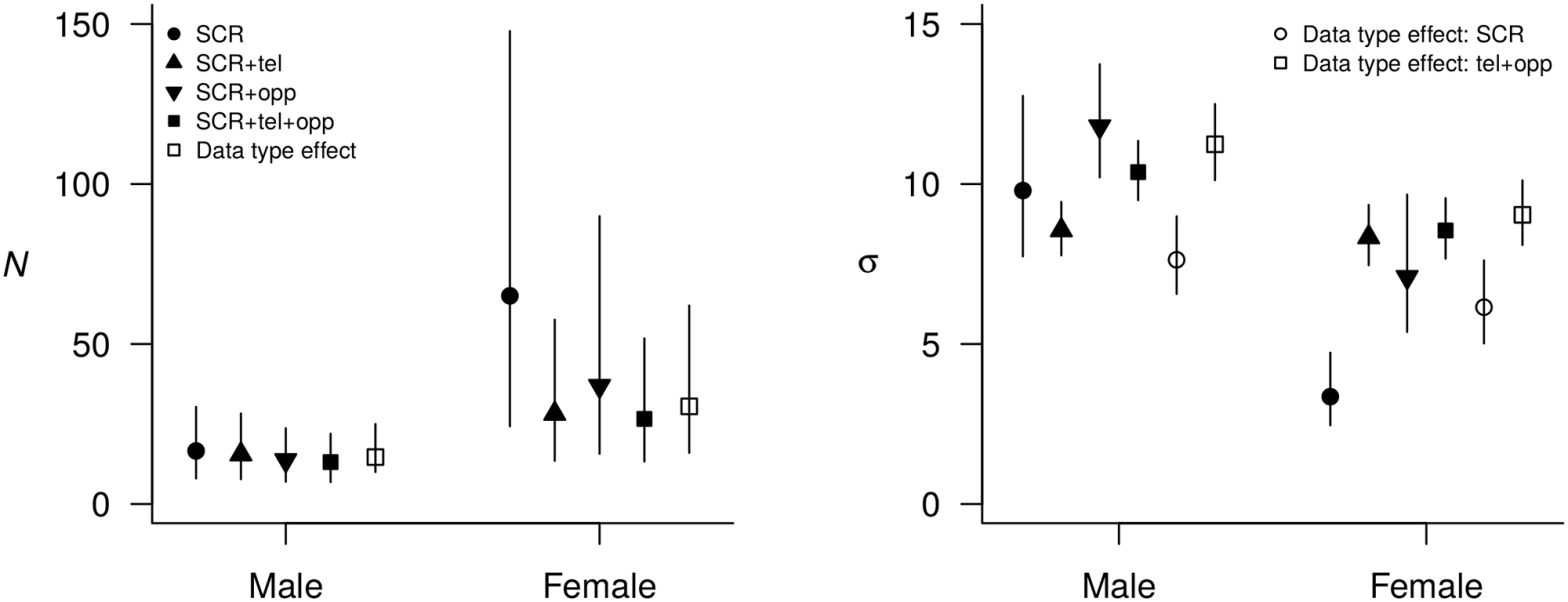
Comparison of posterior estimates for population size (*N*) and spatial scale parameters of the gaussian kernel (*σ*) from the different models. Mean and 95% Bayesian Credible Interval achieved using structured (SCR) data only, or integrating them with unstructured data, i.e. telemetry (‘tel’) and opportunistic (‘opp’) data, available for the brown bear population in the Italian Alps. Filled points correspond to models with implied data consistency, empty points refer to the fully integrated model that account for data inconsistency.

Overall, when consistency was assumed, integrating all available sources of information (traditional SCR, telemetry and opportunistic data) produced more precise estimates of population size and spatial scale parameters when compared to models using either SCR data alone or integrating a single additional data source (Fig 3, Tables 2 and 3). In particular, the gain in precision achieved by jointly modeling all three data types was particularly relevant for sex-specific population size estimates (*N*_sex_). In addition to the increase in precision, integrating additional sources of information resulted in a shift in the median abundance point estimates: from 16 (BCI: 8–30) under the SCR-only model to 13 (BCI: 7–22) under the fully integrated model for males (a 19% difference), and from 58 (BCI: 24–148) to 25 (BCI: 13–52) for females (a 57% difference).

**Table 2:** Posterior estimates of population size achieved using structured (SCR) data alone or integrated with unstructured (telemetry and opportunistic; tel, opp) information available for the brown bear population in the Italian Alps. Parameters estimates are reported for males (*N*_m_) and females (*N*_f_).

Model	Parameter	mean	SD	2.5%	50%	97.5%
SCR	*N*_m_	16.574	5.766	8.000	16.000	30.000
	*N*_f_	65.057	31.086	24.000	58.000	148.000
		data consistency implied				
SCR + tel	*N*_m_	15.514	5.297	8.000	15.000	28.000
	*N*_f_	28.206	11.284	14.000	26.000	58.000
SCR + opp	*N*_m_	13.687	4.297	7.000	13.000	24.000
	*N*_f_	36.890	18.900	16.000	32.000	90.000
SCR + tel + opp	*N*_*m*_	13.088	3.893	7.000	13.000	22.000
	*N*_*f*_	26.587	10.249	13.000	25.000	52.000
		data consistency tested				
SCR + tel + opp	*N*_m_	14.661	4.156	10.000	14.000	25.000
	*N*_f_	30.516	12.341	16.000	28.000	62.000

**Table 3:** Posterior estimates of spatial scale parameter achieved using structured (SCR) data alone or integrated with unstructured (telemetry and opportunistic; tel, opp) information available for the brown bear population in the Italian Alps. Parameters are denoted as follows (m = male, f = female): sex-specific spatial scale parameter shared among different data types, *σ*_sex_; sex-specific spatial scale parameter for structured and unstructured data, *σ*_st,sex_ and *σ*_un,sex_, respectively.

Model	Parameter	mean	SD	2.5%	50%	97.5%
SCR	*σ*_m_	9.796	1.277	7.739	9.644	12.756
	*σ*_f_	3.355	0.582	2.460	3.274	4.729
		data consistency implied				
SCR + tel	*σ*_m_	8.556	0.425	7.773	8.538	9.441
	*σ*_f_	8.339	0.482	7.461	8.316	9.345
SCR + opp	*σ*_m_	11.800	0.905	10.207	11.741	13.743
	*σ*_f_	7.092	1.103	5.380	6.936	9.669
SCR + tel + opp	*σ*_m_	10.372	0.474	9.496	10.351	11.348
	*σ*_f_	8.548	0.481	7.669	8.523	9.558
		data consistency tested				
SCR + tel + opp	*σ*_st,m_	7.632	0.620	6.567	7.582	8.991
	*σ*_un,m_	11.242	0.606	10.121	11.218	12.498
	*σ*_st,f_	6.149	0.667	5.021	6.094	7.612
	*σ*_un,f_	9.037	0.515	8.096	9.014	10.112

Precision gains in estimates of *σ* were minimal when adding opportunistic data (fewest additional data points) and highest when integrating telemetry information only (most additional data points), and was higher for males than females (Fig 3, Table 3). As with the estimates of bear population size, the integration of additional information led to a change in the point estimates of *σ*; compared to the SCR-only model, there was a noticeable increase in the scale of space use when any of the additional data was used. The female 95% home range size estimated from the half normal encounter model under the SCR-only model was 218 *km*^2^ (BCI: 114–421) whereas for the fully integrated model, the estimate was 1379 *km*^2^ (BCI: 1107–1719). Conversely, estimates of male space use, *σ*_*m*_, were consistent across models (Fig 3, Table 3), as were the corresponding 95% home range size estimates: 1836 *km*^2^ (BCI: 1127–3062) under the SCR data only and 2029 *km*^2^ (BCI: 1697–2424) under the fully integrated model.

### Testing and accounting for data inconsistency

There was strong support for the need to account for differences in the spatial scale parameter as estimated from structured *vs*. unstructured data (model 5); posterior inclusion probability of the data-type effect was Pr(*w* = 1) = 0.98. Population size estimates were similar to those obtained with the fully integrated model where data consistency was assumed (14 males, BCI 10–25; 28 females, 16–62; Fig 3, Table 3). The inclusion of a sex-specific data type effect on the spatial scale parameter supported the inconsistency of information on space use provided by structured and unstructured data, with significantly larger *σ* values, and thus larger home range size estimates, derived from unstructured data than those obtained from structured data. Specifically, the difference between data type-specific spatial scale parameters was different from zero (*θ*_2_ = 0.389, BCI: 0.187–0.570; Fig 3, S1 Table). Precision in data type-specific *σ* estimates was similar in both fully integrated models. Sex-specific 95% home range size estimates derived from structured and unstructured data were 1103 *km*^2^ (BCI: 812–1521) and 2385 *km*^2^ (BCI: 1928–2940) for males, and 720 *km*^2^ (BCI: 474–1090) and 1542 *km*^2^ (BCI: 1234–1924) for females, respectively.

## Discussion

We developed a formulation of a spatial capture-recapture model that integrates multiple data sources and, importantly, allows formal testing and accounting for parameter consistency among data types which, as a result, improves inferences about density and space use. We illustrated the approach using empirical data from a reintroduced population of brown bears in the Italian Alps, a low density species of conservation concern for which available data are sparse. Specifically, we jointly analyzed traditional SCR data, collected using systematic sampling of hair traps and rub trees, satellite telemetry data, and opportunistic recovery of biological samples. Comparing estimates from models ranging from traditional SCR to a fully integrated SCR model, we demonstrated that the addition of unstructured data results in increased precision in estimates of population size and space use. Most importantly, we identified and explicitly modeled data-type-specific inconsistency in estimates of spatial model parameters which, if ignored, can produce biased estimates of space use and abundance. Our approach provides a model-based procedure for valid integration of different sources of information in ecological studies of spatial processes.

### Integrating spatial data

Estimates of male population size were stable across all models, with highest precision observed for the fully integrated model, i.e., the model with most spatial data, and lowest for the SCR-only model, which had the fewest spatial locations (Fig 3). For females, estimates of population size from models with additional (unstructured) data were comparable, but were different from the SCR-only estimates, both in terms of precision and point estimates. The addition of telemetry and opportunistic data increased the precision in estimates of female population size when compared to the SCR-only model, but overall, precision was lower than for males.

Although the number of individuals observed for the two sexes was similar (12 females and 10 males), there were fewer spatial recaptures at hair traps or rub trees of females (mean 2.1, min 1, max 4) than of males (mean 8.6, min 1, max 21) (S1 Fig). This suggests that smaller SCR data sets benefit most from the approach that integrates high quality spatial information from even a small number of telemetered or opportunistically observed individuals, or conversely, they may be less stable and more sensitive to combining data. It is encouraging however, that population size estimates based on the two independent unstructured data sources produce similar estimates of abundance, suggesting the addition of opportunistic information is beneficial when consistent with information on space use provided by other unstructured data, such as telemetry. It is important to note, however, that while we provide a much needed framework for evaluating parameter consistency across data types, our approach does not assess parameter sensitivity. The test for consistency is, in our opinion, a welcome first step towards better understanding the consequences of combining multiple data sources [47], and relatively straightforward to implement in practice. Evaluating parameter sensitivity on the other hand would require a full-scale simulation, especially in the current case where the different data types represent the extremes of a spectrum: SCR sampling typically generates sparse spatial information on several-to-many individuals, while telemetry studies typically generate large amounts of fine scale spatial data on relatively few individuals.

Unsurprisingly, the addition of high temporal resolution telemetry data results in a larger precision improvement compared to the relatively sparse opportunistic data (Fig 3). However, the addition of even a few opportunistic locations produces an increase in precision relative to inference from the SCR-only model (Fig 3), suggesting that even in the absence of telemetry data, opportunistic data can be important sources of spatial information. Of course, in our application the true values are unknown and accuracy cannot be evaluated, but our findings are consistent with previous population estimates for the same time period [38], and support the notion that estimated female population size derived from the SCR-only is indeed unrealistically high.

As with abundance, estimates of sex-specific *σ* were more precise under the integrated model (Fig 3). The most notable difference was between estimates of female space use from the three integrated models and the SCR-only model. These differences arise for the larger spatial distribution of telemetry locations compared to hair trap and tree rub (SCR) data, and they are likely related to the link between detector type and behavior. The SCR data were comprised of a few spatially clustered encounters which may be the result of hair deposition patterns related to territoriality, potentially at the core of a home range. Telemetry and opportunistic data, on the other hand, are passive location observations that do not require physical deposition of hair, and that could reflect more general and wider ranging space use (Fig 2b). Estimates of abundance are explicitly linked to estimates of space use, and the difference in the spatial scale parameter estimates, which are more pronounced for females, gives rise to the resulting differences in abundance, but perhaps more importantly, is indicative of potential inconsistency in data-specific parameter estimates. In general, these findings further illustrate the fact that integrated data models allow sex-specific effects on detectability, and consequently abundance, to be modeled, which can often be limited by insufficient observations of one sex or the other when traditional SCR are sparse [48, 49].

### Testing and accounting for data inconsistency

To deal with the apparent sensitivity to the data integration shown by the marked shift in estimates of female population size and spatial scale parameter, we developed a formal model-based test for parameter consistency. Specifically, we used a Bayesian variable selection approach to estimate the degree of support for data-type-specific parameter estimates, and in doing so, estimate the differences in the spatial scale parameter. In practice, this allows for a convenient and formal assessment of the strength of evidence for whether a parameter, *σ* in this case (although other parameters could be tested where appropriate), should be shared across data types or instead be data type-specific.

In our case, the fully integrated model with an effect of data type on *σ* revealed a significant difference in the posterior estimates of the spatial scale parameter, suggesting an underestimation of the home range size using hair trap and tree rub data alone. In fact, home range size estimated from structured data was less than half of the dimension estimated using unstructured information in both sexes. On the other hand, population size estimates did not strongly differ when accounting for data inconsistency in the fully integrated model, although estimates in the latter case were more conservative. These results suggest that careful consideration should be given to the biological interpretation of space use in SCR models because the data are related closely to behavior, e.g., territory marking versus freely roaming. This is likely the case in this study, as suggested by fact that the direction of change in the spatial scale parameter is the same for both sexes.

We suspect that the inconsistencies observed in this study are due to the sampling-behaviour interactions, i.e., the fact that in order to be detected, animals must exhibit a certain behavior, whereas both telemetry data and opportunistic sightings are observation of movement around a home range. Thus, inconsistencies are expected when sampling methods are geared towards different behaviors. Apparent inconsistency may also arise in the presence of transient individuals with non-stationary home ranges, e.g. [50]. Thus, it is clear that while integrated data models are appealing, it is important to acknowledge the underlying biology that gives rise to apparently similar data generated from different sampling schemes.

Testing and accounting for data inconsistency in the same modeling framework used to combine structured and unstructured spatially-referenced data may therefore avoid the risk of spurious estimates of space use. This has important implications in the definition of the study design, since estimates of the spatial scale parameter (and ultimately home range size) are used to calibrate trap spacing and in turn the extent of the trap array [15].

## Conclusion

We provide evidence that integrating SCR, telemetry, and unstructured opportunistic data, by conceptually treating opportunistic records as thinned telemetry data, improves inference precision on abundance and space usage, which are key population-level parameters to inform conservation decisions of elusive and difficult-to-study species. However, care must be taken to assess potential inconsistencies in spatial information provided by the different data sets, where telemetry is both the most informative source of space use but also often available only for a few individuals, whose movement may not be representative of population space use. Understanding how animal density changes in space and how the latter is used is crucial when addressing practical issues in population management and conservation [51]. To this end, the use of opportunistic information increases availability of spatially-referenced individual information, that can be suitably modeled along with other data within a unified framework, thus reducing the need for additional invasive methods. Despite the fact that we can incorporate unstructured data, and thereby increase precision, we should always previously verify whether data are consistent with each other, and thus suitable for integration. The model-based approach here presented offers a natural way of formally testing data consistency.

## Supporting information

S1 Fig Location of bear captures from systematic sampling with hair traps and rub trees (SCR), telemetry and opportunistic records. Grey dots indicate the location of all observed individuals. Bear ID is reported on the top of each plot.
(PDF)

S2 Fig Graphical representation of the data involved in the integrated analysis. Circles represent estimated parameters. Observed data for *n* individuals, detected during *K* visits and members of the population of size *N*, were augmented with *M* − *n* all-zero detections (***Y***_*SCR*_ matrix). SCR data were collected at *J* sites, consisting of *J*_*h*_ hair traps and *J*_*r*_ rub trees. Data set names in Courier font correspond to the names used in the model code. Coordinates, trap deployment, and (standardized) time since last check data sets for the *J* = *J*_*h*_ + *J*_*r*_ traps are denoted by SCR.traps, active, and time.elapsed.sc labels, respectively. Raster data contain information on the distance from the point were founders were released for each of the *nG* pixels (d2core) and was used to model density. Telemetry and opportunistic data were formatted in the same way, with an augmented matrix for number of records available for individual *i* in occasion *k* (n.obs.TEL and n.obs.OPP, respectively) and the x (TEL.y_x, OPP.y_x) and y (TEL.y_y, OPP.y_y) coordinates of those records for each individual in each of the *R*_*tel*_ or *R*_*opp*_ locations and occasion *k*.
(PDF)

S1 Text Details on the Bayesian variable selection. (PDF)

S2 Text R and BUGS codes for the fully integrated models. (PDF)

S1 Table Posterior parameter estimates achieved using structured (SCR) data alone or integrated with unstructured (telemetry and opportunistic; tel, opp) information available for the brown bear population in the Italian Alps. Parameters are denoted as follows: effect of sex on the spatial scale parameter for structured data, *θ*_1_; effect of data type on the spatial scale parameter, *θ*_2_; baseline encounter probability (*p*_0_) intercept, *γ*_0_; effect of being male on *p*_0_, *γ*_1_; effect of trap type on *p*_0_, *γ*_2_; effect of time since last check on *p*_0_, *γ*_3_; density intercept, *β*_0_; effect of distance from the release point on density, *β*_1_; data augmentation parameter, *ψ*; probability of being a male, *ω*_*sex*_.
(PDF)
