# Supplementary material for: Data integration for inference about spatial processes: A model-based approach to test and account for data inconsistency
Source: PLoS One. 2017 Oct 3;12(10):e0185588. doi: 10.1371/journal.pone.0185588 (PMC5626469; doi:10.1371/journal.pone.0185588)
Supplement: S1 Fig — Grey dots indicate the location of all observed individuals. Bear ID is reported on the top of each plot. (PDF) [file pone.0185588.s001.pdf]

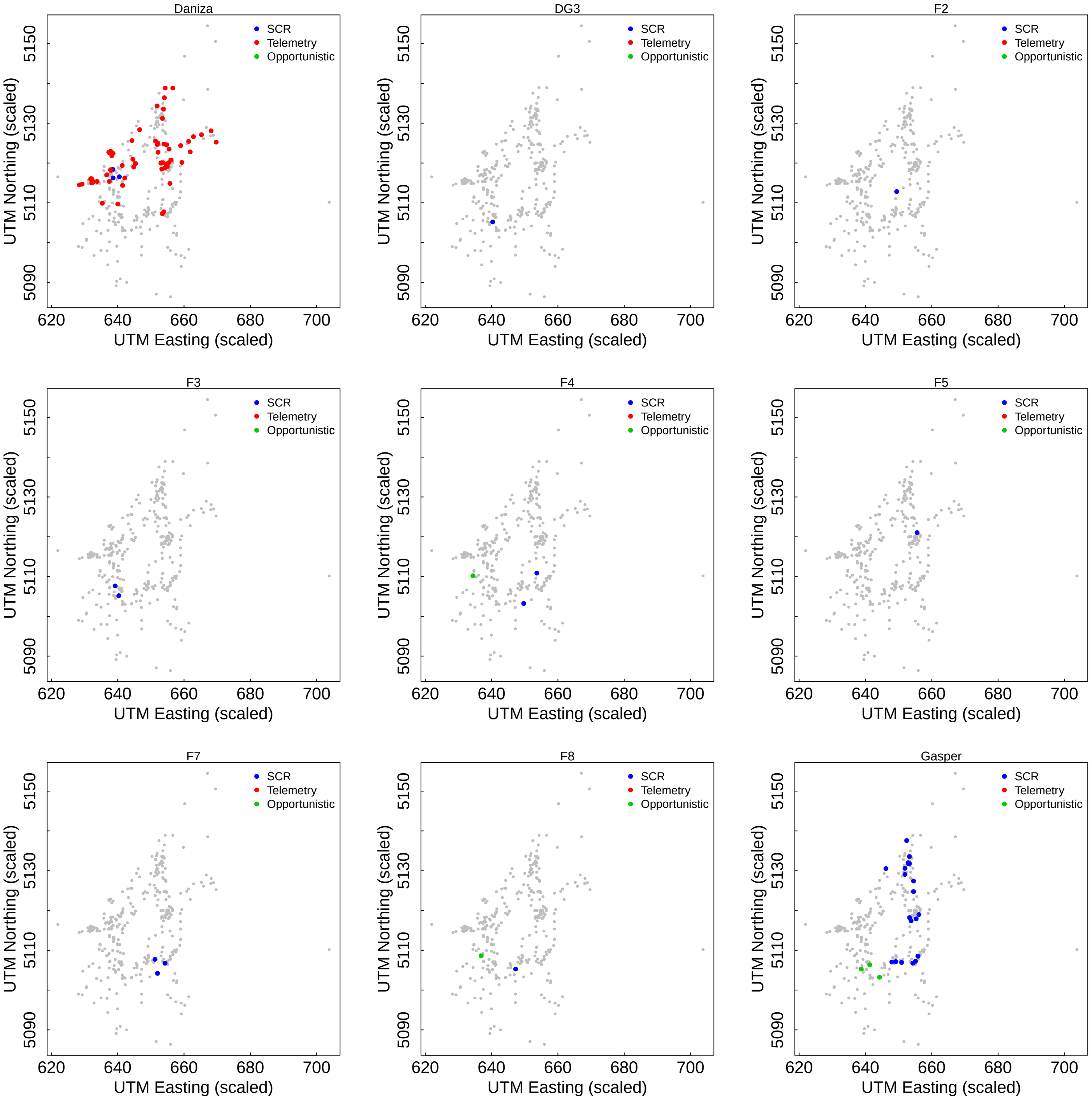

S1 Fig (continue)

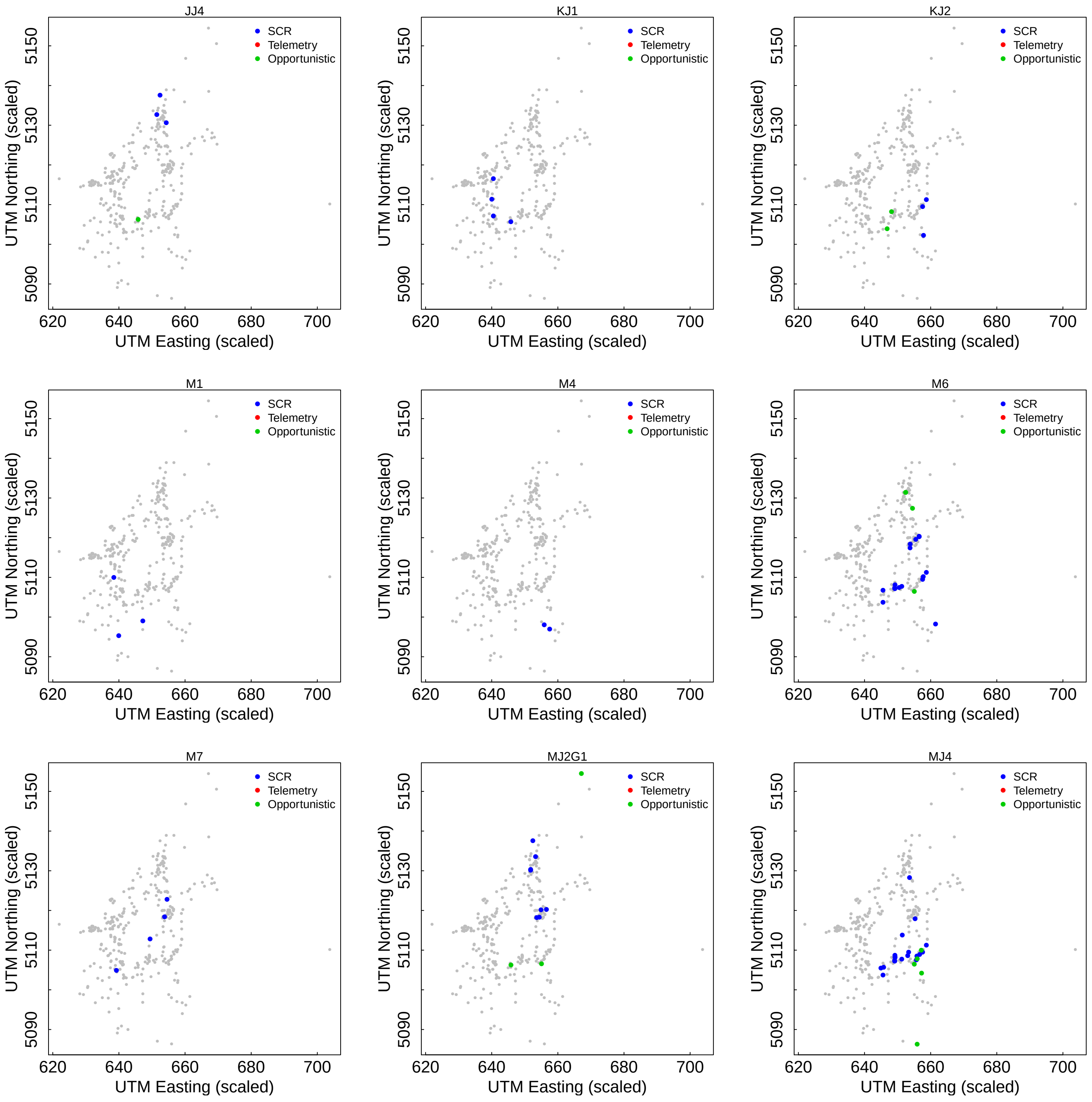

S1 Fig (continue)

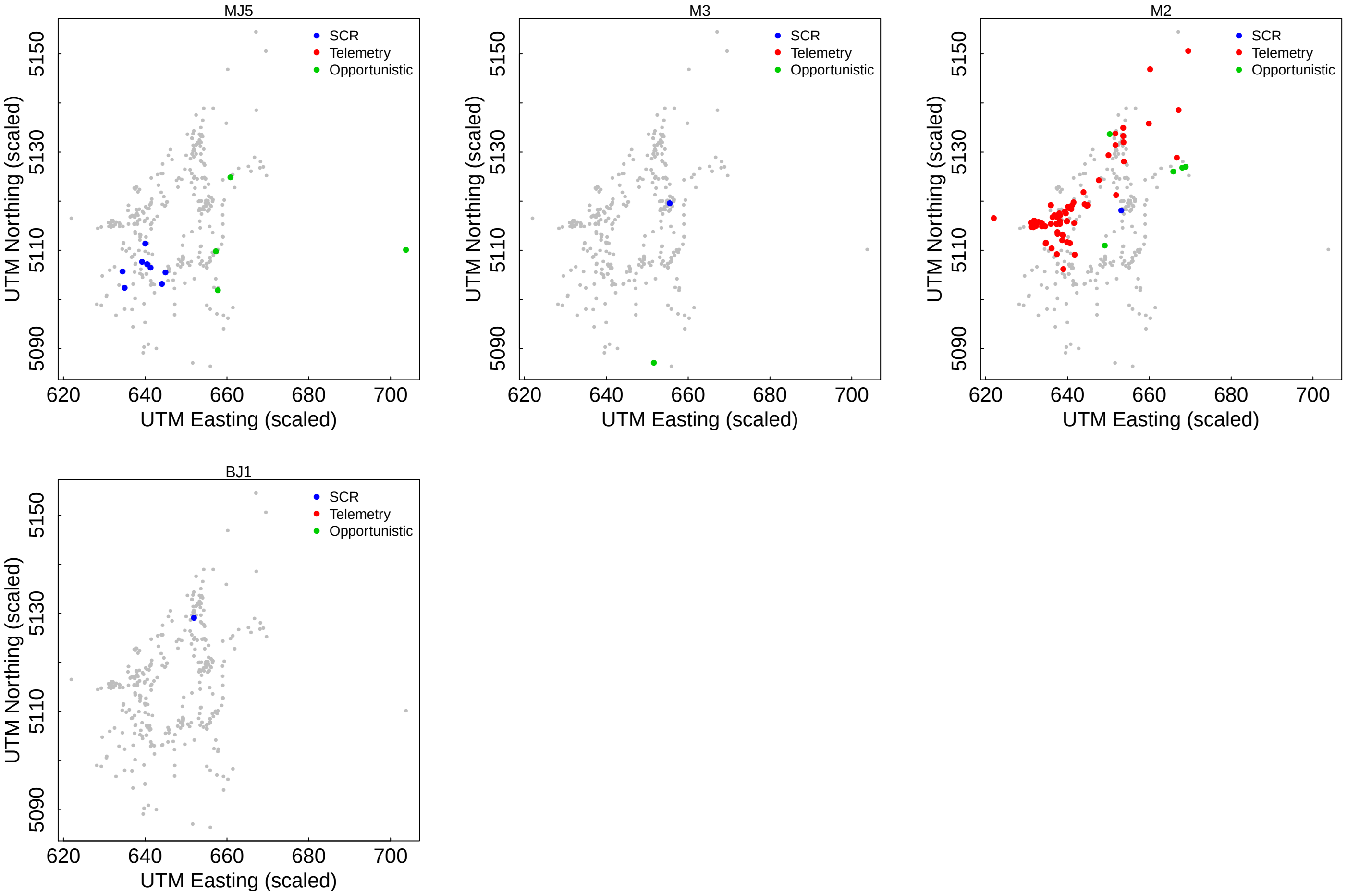

S1 Fig. Location of bear captures from systematic sampling with hair traps and rub trees (SCR), telemetry and opportunistic records. Grey dots indicate the location of all observed individuals. Bear ID is reported on the top of each plot.
