# Supplementary material for: Data integration for inference about spatial processes: A model-based approach to test and account for data inconsistency
Source: PLoS One. 2017 Oct 3;12(10):e0185588. doi: 10.1371/journal.pone.0185588 (PMC5626469; doi:10.1371/journal.pone.0185588)
Supplement: S2 Fig — Circles represent estimated parameters. Observed data for n individuals, detected during K visits and members of the population of size N, were augmented with M − n all-zero detections (YSCR matrix). SCR data were collected at J sites, consisting of Jh hair traps and Jr rub trees. Data set names in Courier font correspond to the names used in the model code. Coordinates, trap deployment, and (standardized) time since last check data sets for the J = Jh + Jr traps are denoted by SCR.traps, active, and time.elapsed.sc labels, respectively. Raster data contain information on the distance from the point were founders were released for each of the nG pixels (d2core) and was used to model density. Telemetry and opportunistic data were formatted in the same way, with an augmented matrix for number of records available for individual i in occasion k (n.obs.TEL and n.obs.OPP, respectively) and the x (TEL.y_x, OPP.y_x) and y (TEL.y_y, OPP.y_y) coordinates of those records for each individual in each of the Rtel or Ropp locations and occasion k. (PDF) [file pone.0185588.s002.pdf]

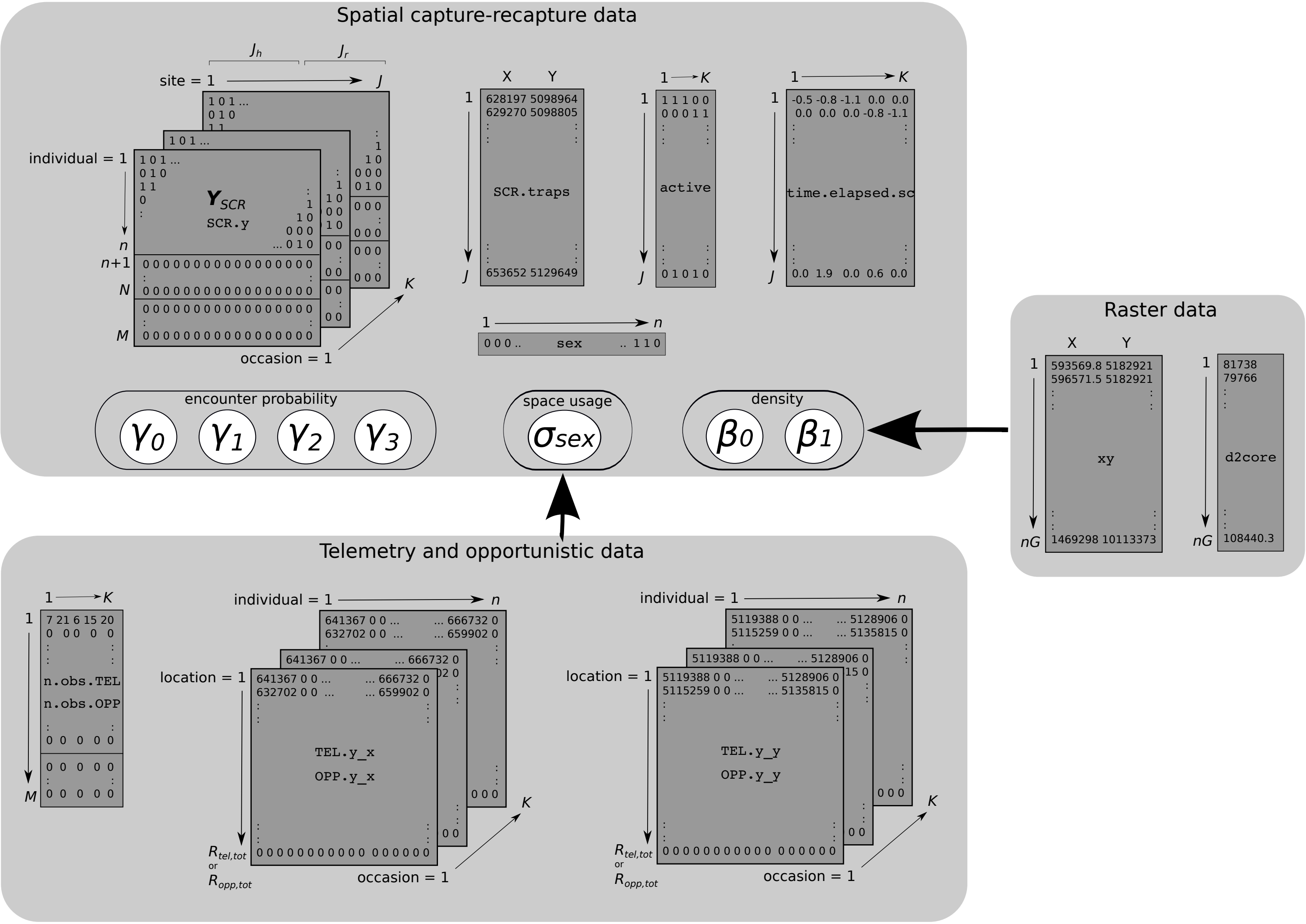

S2 Fig. Graphical representation of the data involved in the integrated analysis. Circles represent estimated parameters. Observed data for n individuals, detected during K visits and members of the population of size N, were augmented with $M - n$ all-zero detections ($\boldsymbol{Y}_{SCR}$ matrix). SCR data were collected at J sites, consisting of J_h hair traps and J_r rub trees. Data set names in Courier font correspond to the names used in the model code. Coordinates, trap deployment, and (standardized) time since last check data sets for the $J = J_h + J_r$ traps are denoted by `SCR.traps`, `active`, and `time.elapsed.sc` labels, respectively. Raster data contain information on the distance from the point were founders were released for each of the nG pixels (`d2core`) and was used to model density. Telemetry and opportunistic data were formatted in the same way, with an augmented matrix for number of records available for individual i in occasion k (`n.obs.TEL` and `n.obs.OPP`, respectively) and the x (`TEL.y_x`, `OPP.y_x`) and y (`TEL.y_y`, `OPP.y_y`) coordinates of those records for each individual in each of the R_{tel} or R_{opp} locations and occasion k.
