# Supplementary material for: Data integration for inference about spatial processes: A model-based approach to test and account for data inconsistency
Source: PLoS One. 2017 Oct 3;12(10):e0185588. doi: 10.1371/journal.pone.0185588 (PMC5626469; doi:10.1371/journal.pone.0185588)
Supplement: S1 Text — (PDF) [file pone.0185588.s003.pdf]

S1 Text. Details on the Bayesian variable selection

Prior probability for θ_2 was specified as a mixture of normal distributions:

$$\Pr(\theta_2|w) = (1 - w)\,\text{Normal}(0, 1) + w\,\text{Normal}(0, \Sigma)$$

where the fixed prior variance $\Sigma = V/(l + 1)$, with l denoting the number of regressors in Eq. 8 in the main text ($l = 1$ if $w = 0$, and $l = 2$ if $w = 1$) and the total variance in the linear predictor V with a Gamma(3.2890,7.8014) prior assigned [1].

REFERENCES

1. Link WA, Barker RJ. Model weights and the fundations of multimoldel inference. Ecology. 2006;87(10):2626–2635.
