# Supplementary material for: Data integration for inference about spatial processes: A model-based approach to test and account for data inconsistency
Source: PLoS One. 2017 Oct 3;12(10):e0185588. doi: 10.1371/journal.pone.0185588 (PMC5626469; doi:10.1371/journal.pone.0185588)
Supplement: S2 Text — (PDF) [file pone.0185588.s004.pdf]

# S2 Text. R and BUGS codes for the fully integrated models

We provide the code to fit the models where: (i) data consistency is assumed, (ii) data consistency is tested, (iii) data inconsistency is accounted for.

```
#################################################################
#------ 1) Fully integrated model: data consistency assumed ------#
#################################################################
cat("
model{
### PRIORS
  for(cv in 1:4){ # detection coefficients
    p.b[cv] ~ dnorm(0,0.01)
  }
  for(cv in 1:2){ # density coefficients
    d.b[cv] ~ dnorm(0,0.01)
  }
### Density
  for(np in 1:nPix){
    mu.d[np] <- exp(d.b[1] +
                    d.b[2] * d2core[np])
    probs[np] <- mu.d[np] / EN
  }
  EN <- sum(mu.d[])
  psi <- EN/M
### SIGMA
  for(sx in 1:2){ # sex-specific sigma
    a1[sx] ~ dnorm(0,.1)
    sigma[sx] <- sqrt(1/(2*a1[sx]))
  }
### SEX
  psi.sex ~ dunif(0,1)
### LIKELIHOOD
  for(i in 1:M){ # individuals
    z[i] ~ dbern(psi)
    sex[i] ~ dbern(psi.sex) # 0=female
    pick.s[i] ~ dcat(probs[])
    s[i,1] <- G[pick.s[i],1]
    s[i,2] <- G[pick.s[i],2]
    for(k in 1:K){ # occasions
      # SCR observations (hair traps and rub trees)
```

```
   for(j in 1:ntraps){
    d[i,j,k] <- sqrt(pow(s[i,1] - scr.locs[j,1],2) + pow(s[i,2] - scr.locs[j,2],2))
    logit(p0[i,j,k]) <- (p.b[1] +
               p.b[2] * sex[i] +
               p.b[3] * trap.type[j] +
               p.b[4] * time.elapsed[j,k])
    p[i,j,k] <- p0[i,j,k] * exp(-a1[sex[i]+1] * d[i,j,k]*d[i,j,k])
    mu.p[i,j,k] <- z[i] * p[i,j,k] * active[j,k]
    scrY[i,j,k] ~ dbern(mu.p[i,j,k])
  }
   # TELEMETRY observations
   for(tel in 1:n.obs.TEL[i,k]){
    telY.x[tel,i,k] ~ dnorm(s[i,1], 1/(sigma[sex[i]+1]^2))
    telY.y[tel,i,k] ~ dnorm(s[i,2], 1/(sigma[sex[i]+1]^2))
   }
   # OPPORTUNISTIC observations
   for(opp in 1:n.obs.OPP[i,k]){
    oppY.x[opp,i,k] ~ dnorm(s[i,1], 1/(sigma[sex[i]+1]^2))
    oppY.y[opp,i,k] ~ dnorm(s[i,2], 1/(sigma[sex[i]+1]^2))
   }
 }} #k & i
### Derive population size (N) density (D)
 N <- sum(z[])
 D <- N/area
 N_female <- N - N_male
 D_female <- N_female/area
 N_male <- sum(z[]*sex[])
 D_male <- N_male/area
}
",file="integratedSCR.txt")


##################################################################
#--- 2) Fully integrated model: testing for data consistency ----#
##################################################################
cat("
model{
### PRIORS
 for(cv in 1:4){ # detection coefficients
  p.b[cv] ~ dnorm(0,0.01)
```

```
  }
  for(cv in 1:2){ # density coefficients
    d.b[cv] ~ dnorm(0,0.01)
  }
### Density
  for(np in 1:nPix){
    mu.d[np] <- exp(d.b[1] +
                    d.b[2] * d2core[np])
    probs[np] <- mu.d[np] / EN
  }
  EN <- sum(mu.d[])
  psi <- EN/M
### SIGMA
  int.sigma ~ dunif(0,15)
  beta.sex.hair ~ dunif(-3,3)

  # Prior for data type effect
  predictorsIn <- 1 + w
  beta.type ~ dnorm(0,psuedoTau)T(-3,3)
  tauV ~ dgamma(3.2890,7.8014)
  psuedoTau <- ifelse(w==1,
                    1/((1/tauV) / predictorsIn),
                    0.01*100)
  # Priors for variable indicators
  w ~ dbern(0.5)
### SEX
  psi.sex ~ dunif(0,1)
### LIKELIHOOD
  for(i in 1:M){ # individuals
    z[i] ~ dbern(psi)
    sex[i] ~ dbern(psi.sex) # 0 = female
    pick.s[i] ~ dcat(probs[])
    s[i,1] <- G[pick.s[i],1]
    s[i,2] <- G[pick.s[i],2]
    log(sigma_hair[i]) <- log(int.sigma) + beta.sex.hair * sex[i]
    log(sigma_telopp[i]) <- log(sigma_hair[i]) + w * beta.type
    a1[i] <- 1/(2*sigma_hair[i]*sigma_hair[i])
    for(k in 1:K){ # occasions
      # SCR observations (hair traps and rub trees)
      for(j in 1:ntraps){
```

```
        d[i,j,k] <- sqrt( pow(s[i,1] - scr.locs[j,1],2) + pow(s[i,2] - scr.locs[j,2],2) )
        logit(p0[i,j,k]) <- (p.b[1] +
                             p.b[2] * sex[i] +
                             p.b[3] * trap.type[j] +
                             p.b[4] * time.elapsed[j,k])
        p[i,j,k] <- p0[i,j,k] * exp(-a1[i] * d[i,j,k]*d[i,j,k])
        mu.p[i,j,k] <- z[i] * p[i,j,k] * active[j,k]
        scrY[i,j,k] ~ dbern(mu.p[i,j,k])
      }
       # TELEMETRY observations
       for(tel in 1:n.obs.TEL[i,k]){
         telY.x[tel,i,k] ~ dnorm(s[i,1], 1/(sigma_telopp[i]^2))
         telY.y[tel,i,k] ~ dnorm(s[i,2], 1/(sigma_telopp[i]^2))
       }
       # OPPORTUNISTIC observations
       for(opp in 1:n.obs.OPP[i,k]){
         oppY.x[opp,i,k] ~ dnorm(s[i,1], 1/(sigma_telopp[i]^2))
         oppY.y[opp,i,k] ~ dnorm(s[i,2], 1/(sigma_telopp[i]^2))
       }
  }} #k & i
### Derive population size (N) density (D)
  N <- sum(z[])
  D <- N/area
  N_female <- N - N_male
  D_female <- N_female/area
  N_male <- sum(z[]*sex[])
  D_male <- N_male/area
}
",file="integratedSCR_GVS.txt")



##########################################################################
#---- 3) Fully integrated model: accounting for data inconsistency ----#
##########################################################################
cat("
model{
### PRIORS
  for(cv in 1:4){ # detection coefficients
    p.b[cv] ~ dnorm(0,0.01)
```

```
  }
  for(cv in 1:2){ # density coefficients
    d.b[cv] ~ dnorm(0,0.01)
  }
### Density
  for(np in 1:nPix){
    mu.d[np] <- exp(d.b[1] +
                    d.b[2] * d2core[np])
    probs[np] <- mu.d[np] / EN
  }
  EN <- sum(mu.d[])
  psi <- EN/M
### SIGMA
    int.sigma ~ dunif(0,15)
    beta.sex.hair ~ dunif(-3,3)
    beta.type[sx] ~ dunif(-3,3)
### SEX
  psi.sex ~ dunif(0,1)
### LIKELIHOOD
  for(i in 1:M){ # individuals
    z[i] ~ dbern(psi)
    sex[i] ~ dbern(psi.sex) # 0 is female
    pick.s[i] ~ dcat(probs[])
    s[i,1] <- G[pick.s[i],1]
    s[i,2] <- G[pick.s[i],2]
    log(sigma_hair[i]) <- log(int.sigma) + beta.sex.hair * sex[i]
    log(sigma_telopp[i]) <- log(sigma_hair[i]) + beta.type
    a1[i] <- 1/(2*sigma_hair[i]*sigma_hair[i])
    for(k in 1:K){ # occasions
      # SCR observations (hair traps and rub trees)
      for(j in 1:ntraps){
        d[i,j,k] <- sqrt( pow(s[i,1] - scr.locs[j,1],2) + pow(s[i,2] - scr.locs[j,2],2) )
        logit(p0[i,j,k]) <- (p.b[1] +
                             p.b[2] * sex[i] +
                             p.b[3] * trap.type[j] +
                             p.b[4] * time.elapsed[j,k])
        p[i,j,k] <- p0[i,j,k] * exp(-a1[i] * d[i,j,k]*d[i,j,k])
        mu.p[i,j,k] <- z[i] * p[i,j,k] * active[j,k]
        scrY[i,j,k] ~ dbern(mu.p[i,j,k])
     }
```

```
      # TELEMETRY observations
      for(tel in 1:n.obs.TEL[i,k]){
        telY.x[tel,i,k] ~ dnorm(s[i,1], 1/(sigma_telopp[i]^2))
        telY.y[tel,i,k] ~ dnorm(s[i,2], 1/(sigma_telopp[i]^2))
      }
      # OPPORTUNISTIC observations
      for(opp in 1:n.obs.OPP[i,k]){
        oppY.x[opp,i,k] ~ dnorm(s[i,1], 1/(sigma_telopp[i]^2))
        oppY.y[opp,i,k] ~ dnorm(s[i,2], 1/(sigma_telopp[i]^2))
      }
  }} #k & i
### Derive population size (N) density (D)
  N <- sum(z[])
  D <- N/area
  N_female <- N - N_male
  D_female <- N_female/area
  N_male <- sum(z[]*sex[])
  D_male <- N_male/area
}
",file="integratedSCR_2.txt")


##############################################################
#--------- start part in common to all models --------------#
##############################################################
# Load R packages
library(scrbook)
library(secr)
library(car)
library(jagsUI)
library(abind)
# Starting data
load("bear_data_Tenan_et_al.RData")
# Data Augmentation
M <- 200
nind <- nrow(SCR.y)
aug <- M-nind
ntraps <- dim(SCR.y)[2]
K <- dim(SCR.y)[3]
```

```
SCR.y <- abind(SCR.y,array(0,c(aug,ntraps,K)),along=1)
SCR.y[SCR.y>0] <- 1
sex <- c(sex,rep(NA,aug))
active <- ifelse(is.na(time.elapsed[,-1]),0,1)
time.elapsed.sc <- time.elapsed[,-1]
time.elapsed.sc[] <- scale(unlist(c(time.elapsed.sc)))
time.elapsed.sc[is.na(time.elapsed.sc)] <- 0
n.obs.TEL <- rbind(n.obs.TEL,matrix(0,aug,K))
n.obs.OPP <- rbind(n.obs.OPP,matrix(0,aug,K))
# Manipulate the state space
all.obs <- cbind(c(SCR.traps[,2],c(TEL.y_x[TEL.y_x!=0]),c(OPP.y_x[OPP.y_x!=0])),
                 c(SCR.traps[,3],c(TEL.y_y[TEL.y_y!=0]),c(OPP.y_y[OPP.y_y!=0])))
cut.S <- apply(e2dist(xy,all.obs),1,min)<=21000
xy.cut <- xy[cut.S,]
zz <- ifelse(!is.na(sex),1,NA)
# Data
bugs.data <- list(scrY = SCR.y,
                  telY.x = TEL.y_x/1000,
                  telY.y = TEL.y_y/1000,
                  oppY.x = OPP.y_x/1000,
                  oppY.y = OPP.y_y/1000,
                  M = M, K = K,
                  ntraps = ntraps,
                  area = nrow(xy)*4,#km^2
                  scr.locs = SCR.traps[,-1]/1000,
                  n.obs.TEL = n.obs.TEL,
                  n.obs.OPP = n.obs.OPP,
                  sex = sex,
                  time.elapsed = time.elapsed.sc,
                  trap.type = trap.type,
                  active = active,
                  G = xy[cut.S,]/1000,
                  nPix = nrow(xy[cut.S,]),
                  d2core = as.numeric(scale(d2core[cut.S])))
# Initial values function
init.pick.s <- numeric(M)
for(i in 1:nind){
    if (sum(bugs.data$scrY[i,,]) == 0)
        next
    tmpDmat <- e2dist(bugs.data$G,
```

```
                      cbind(
                      rep(bugs.data$scr.locs[,1], apply(bugs.data$scrY[i,, ],1,sum)),
                      rep(bugs.data$scr.locs[,2], apply(bugs.data$scrY[i,, ],1,sum))
                           )
                     )
    tmp.s <- which.min(apply(tmpDmat,1,min))
    init.pick.s[i] <- tmp.s
}
init.pick.s[init.pick.s==0] <- sample(sum(init.pick.s==0),1:nrow(xy[cut.S,]))
zst <- c(rep(NA, nind), rbinom(aug, 1, 0.5))
#############################################################
#----------- end part in common to all models --------------#
#############################################################


###############################################################
#--------- 1) Fit model with data consistency assumed --------#
###############################################################
# initial values
inits <- function() {
    list(psi.sex=runif(0.5),
         sex=c(rep(NA,nind),rbinom(aug,1,0.5)),
         a1= c(0.007,0.005),
         p.b = c(-1,0.1,-0.1,0.1),
         d.b = c(-6,0),
         pick.s = init.pick.s,
         z = c(rep(1,bugs.data$M)))
}
# parameters to monitor
parameters<-c("N_male","N_female","sigma","p.b","d.b", "psi","psi.sex")
# MCMC settings
n.adapt  <-  1000
n.burnin <-  2000
n.iter   <- 30000
thin     <-     1
chains   <-     3
# Fit the model
out.all <- jags(data = bugs.data,
                inits = inits,
                parameters.to.save = parameters,
```

```
                model.file = "integratedSCR.txt",
                n.chains = chains,
                n.adapt = n.adapt,
                n.iter = n.iter,
                n.burnin = n.burnin,
                n.thin = thin,
                parallel=T)


################################################################
#--------- 2) Fit model for testing data consistency ---------#
################################################################
# initial values
inits <- function() {
    list(psi.sex=runif(0.5),
         sex=c(rep(NA,nind),rbinom(aug,1,0.5)),
         tauV = runif(1,0.1,1.1),
         int.sigma=runif(1,5,8),beta.sex.hair=0,beta.type=0,
         p.b = c(-1,0.1,-0.1,0.1),
         d.b = c(-6,0),
         pick.s = init.pick.s,
         z = c(rep(1,bugs.data$M)))
}
# parameters to monitor
parameters<-c("N_male","N_female","int.sigma","beta.sex.hair",
              "beta.sex.telopp","beta.type","p.b","d.b",
              "psi","psi.sex","w")
# MCMC settings
n.adapt  <-  1000
n.burnin <-  2000
n.iter   <- 30000
thin     <-     1
chains   <-     3
# Fit the model
out.all <- jags(data = bugs.data,
                inits = inits,
                parameters.to.save = parameters,
                model.file = "integratedSCR_GVS.txt",
                n.chains = chains,
                n.adapt = n.adapt,
```

```
                n.iter = n.iter,
                n.burnin = n.burnin,
                n.thin = thin,
                parallel=T)


################################################################
#------- 3) Fit model accounting for data inconsistency ------#
################################################################
# initial values
inits <- function() {
    list(psi.sex=runif(0.5),
         sex=c(rep(NA,nind),rbinom(aug,1,0.5)),
         int.sigma=runif(1,5,8),beta.sex.hair=0,beta.type=0,
         p.b = c(-1,0.1,-0.1,0.1),
         d.b = c(-6,0),
         pick.s = init.pick.s,
         z = c(rep(1,bugs.data$M)))
}
# parameters to monitor
parameters<-c("N_male","N_female","int.sigma","beta.sex.hair",
              "beta.sex.telopp","beta.type",
              "p.b","d.b", "psi","psi.sex")
# MCMC settings
n.adapt  <-  1000
n.burnin <-  2000
n.iter   <- 30000
thin     <-     1
chains   <-     3
# Fit the model
out.all <- jags(data = bugs.data,
                inits = inits,
                parameters.to.save = parameters,
                model.file = "integratedSCR_2.txt",
                n.chains = chains,
                n.adapt = n.adapt,
                n.iter = n.iter,
                n.burnin = n.burnin,
                n.thin = thin,
                parallel=T)
```
