# Supplementary material for: Data integration for inference about spatial processes: A model-based approach to test and account for data inconsistency
Source: PLoS One. 2017 Oct 3;12(10):e0185588. doi: 10.1371/journal.pone.0185588 (PMC5626469; doi:10.1371/journal.pone.0185588)
Supplement: S1 Table — Parameters are denoted as follows: effect of sex on the spatial scale parameter for structured data, θ1; effect of data type on the spatial scale parameter, θ2; baseline encounter probability (p0) intercept, γ0; effect of being male on p0, γ1; effect of trap type on p0, γ2; effect of time since last check on p0, γ3; density intercept, β0; effect of distance from the release point on density, β1; data augmentation parameter, ψ; probability of being a male, ωsex. (PDF) [file pone.0185588.s005.pdf]

S1 Table. Posterior parameter estimates achieved using structured (SCR) data alone or integrated with unstructured (telemetry and opportunistic; tel, opp) information available for the brown bear population in the Italian Alps. Parameters are denoted as follows: effect of sex on the spatial scale parameter for structured data, θ_1; effect of data type on the spatial scale parameter, θ_2; baseline encounter probability (p_0) intercept, γ_0; effect of being male on p_0, γ_1; effect of trap type on p_0, γ_2; effect of time since last check on p_0, γ_3; density intercept, β_0; effect of distance from the release point on density, β_1; data augmentation parameter, ψ; probability of being a male, ω_{sex}.

	SCR + tel + opp (data consistency tested)				
Parameter	mean	SD	2.5%	50%	97.5%
θ_1	0.218	0.075	0.070	0.219	0.364
θ_2	0.389	0.098	0.187	0.393	0.570
γ_0	-3.246	0.339	-3.927	-3.237	-2.600
γ_1	1.572	0.315	0.976	1.565	2.206
γ_2	-1.254	0.395	-2.024	-1.256	-0.474
γ_3	0.652	0.189	0.274	0.652	1.020
β_0	-3.196	0.748	-4.802	-3.147	-1.874
β_1	-1.196	0.491	-2.210	-1.176	-0.286
ψ	0.225	0.080	0.118	0.209	0.425
ω_{sex}	0.343	0.096	0.174	0.336	0.547
	SCR + tel + opp (data consistency implied)				
γ_0	-3.714	0.308	-4.335	-3.705	-3.125
γ_1	1.623	0.312	1.024	1.618	2.246
γ_2	-1.153	0.387	-1.899	-1.155	-0.384
γ_3	0.619	0.185	0.248	0.621	0.976
β_0	-3.245	0.670	-4.719	-3.192	-2.081
β_1	-1.128	0.467	-2.125	-1.107	-0.276
ψ	0.198	0.064	0.109	0.186	0.351
ω_{sex}	0.341	0.096	0.171	0.335	0.542
	SCR + opp (data consistency implied)				
γ_0	-3.414	0.345	-4.116	-3.406	-2.758
γ_1	1.417	0.360	0.734	1.409	2.143
γ_2	-1.160	0.392	-1.923	-1.164	-0.390
γ_3	0.621	0.189	0.248	0.623	0.985
β_0	-2.713	0.600	-3.960	-2.687	-1.562
β_1	-0.739	0.435	-1.620	-0.731	0.113
ψ	0.252	0.106	0.124	0.227	0.542
ω_{sex}	0.291	0.096	0.125	0.285	0.498
	SCR + tel (data consistency implied)				
γ_0	-3.479	0.332	-4.139	-3.478	-2.833
γ_1	1.791	0.343	1.119	1.787	2.472
γ_2	-1.207	0.402	-1.991	-1.207	-0.413
γ_3	0.670	0.193	0.288	0.671	1.046
β_0	-2.956	0.627	-4.313	-2.899	-1.869
β_1	-0.890	0.460	-1.842	-0.866	-0.043
ψ	0.218	0.074	0.115	0.204	0.406
ω_{sex}	0.364	0.098	0.187	0.359	0.569
	SCR				
γ_0	-2.220	0.407	-3.040	-2.213	-1.451
γ_1	0.804	0.441	-0.046	0.799	1.683
γ_2	-1.207	0.421	-2.037	-1.204	-0.389
γ_3	0.658	0.203	0.263	0.656	1.060
β_0	-2.076	0.549	-3.259	-2.032	-1.119
β_1	-0.459	0.387	-1.267	-0.437	0.244
ψ	0.406	0.168	0.177	0.371	0.850
ω_{sex}	0.223	0.084	0.090	0.212	0.414
